# Cell Surface Markers in HTLV-1 Pathogenesis

**DOI:** 10.3390/v3081439

**Published:** 2011-08-16

**Authors:** Andrea K. Kress, Ralph Grassmann, Bernhard Fleckenstein

**Affiliations:** Institute of Clinical and Molecular Virology, Friedrich-Alexander-Universität Erlangen-Nürnberg, Schlossgarten 4, 91054 Erlangen, Germany; E-Mail: Bernhard.Fleckenstein@viro.med.uni-erlangen.de (B.F.)

**Keywords:** HTLV-1, Tax, oncoprotein, phenotype, differentiation, ATLL, HAM/TSP, TNFR, cytokine receptor, interleukin

## Abstract

The phenotype of HTLV-1-transformed CD4^+^ T lymphocytes largely depends on defined viral effector molecules such as the viral oncoprotein Tax. In this review, we exemplify the expression pattern of characteristic lineage markers, costimulatory receptors and ligands of the tumor necrosis factor superfamily, cytokine receptors, and adhesion molecules on HTLV-1-transformed cells. These molecules may provide survival signals for the transformed cells. Expression of characteristic surface markers might therefore contribute to persistence of HTLV-1-transformed lymphocytes and to the development of HTLV-1-associated disease.

## 1. Pathogenetic Properties of Human T Cell Lymphotropic Virus Type 1 (HTLV-1)

Human T cell lymphotropic virus type 1 (HTLV-1), a delta-retrovirus, is the causative agent of a severe and fatal lymphoproliferative disorder of CD4^+^ T cells, adult T cell leukemia/lymphoma (ATLL), and of a neurodegenerative, inflammatory disease, HTLV-1-associated myelopathy/tropical spastic paraparesis (HAM/TSP) [1–5]. Both diseases can develop as a consequence of prolonged viral persistence in T cells after a latency of decades. The risk of developing ATLL among virus carriers is estimated to be 6.6% for males and 2.1% for females, while 1–4% of the infected individuals may develop HAM/TSP [6,7]. HTLV-1 has developed a unique strategy for lifelong persistence in the presence of an active immune system. This is achieved by replication of the virus mainly in its provirus form, stimulation of cell division by the virus, and, as a consequence, clonal amplification of infected cells. Virus-infected clones are detectable and can persist over many years even in non-leukemic individuals [8]. The stimulation of T cell proliferation in patients by viral gene expression was corroborated by cell dynamic studies which revealed a correlation of the *in vivo* proliferation rate of CD4^+^CD45R0^+^ cells, the main cell type infected with HTLV-1 *in vivo* [9], with viral expression *ex vivo* [10].

## 2. Viral Effector Molecules

Upon infection, HTLV-1 integrates into the host cell genome and is mainly maintained in its provirus form (9.1 kb) which is flanked by long terminal repeats (LTR) in both the 5′ and 3′ region. In addition to structural proteins common for retroviruses, protease, and reverse transcriptase, HTLV-1 encodes accessory and regulatory proteins [6]. While the accessory proteins p12, p30, p13 and HBZ are important for viral infectivity and replication, they are not required for lymphocyte immortalization [11,12]. HBZ, which is transcribed as an antisense transcript of HTLV-1 from the 3′ LTR, promotes proliferation of ATLL cells [13]. The regulatory proteins Tax and Rex are both essential for viral replication [14]. While Tax strongly enhances viral mRNA synthesis by transactivating the HTLV-1-LTR promoter, Rex controls the synthesis of the structural proteins on a posttranscriptional level [15,16].

Tax confers transforming properties on HTLV-1, as it can immortalize primary human T cells [17–19], and induce leukemia in transgenic mice [20]. Several Tax functions may contribute to its transforming capacity, including interference with cell cycle check points, tumor suppressors and DNA repair. To promote cell proliferation, Tax can stimulate the expression of cellular proteins controlling proliferation and survival [21–23]. Beyond that, Tax induces cellular genes which may contribute to HTLV-1-mediated pathogenesis such as the tumor marker and actin-bundling protein Fascin [24]. Tax is capable of stimulating cellular transcription by interacting with various signaling pathways such as both the canonical and non-canonical nuclear factor kappa B (NF-κB) pathways [21,22,25,26], cAMP response element-binding protein (CREB) and serum response factor (SRF) pathways [14,27]. In the early phase of tumor progression in patients, Tax is required to initiate transformation. By contrast, Tax is no longer expressed in many ATLL-cells in late stages of tumor progression, while Tax-induced signaling pathways are still maintained [28].

## 3. Differentiation of Human T Lymphocytes and HTLV-1-Persistence

**Figure 1.**
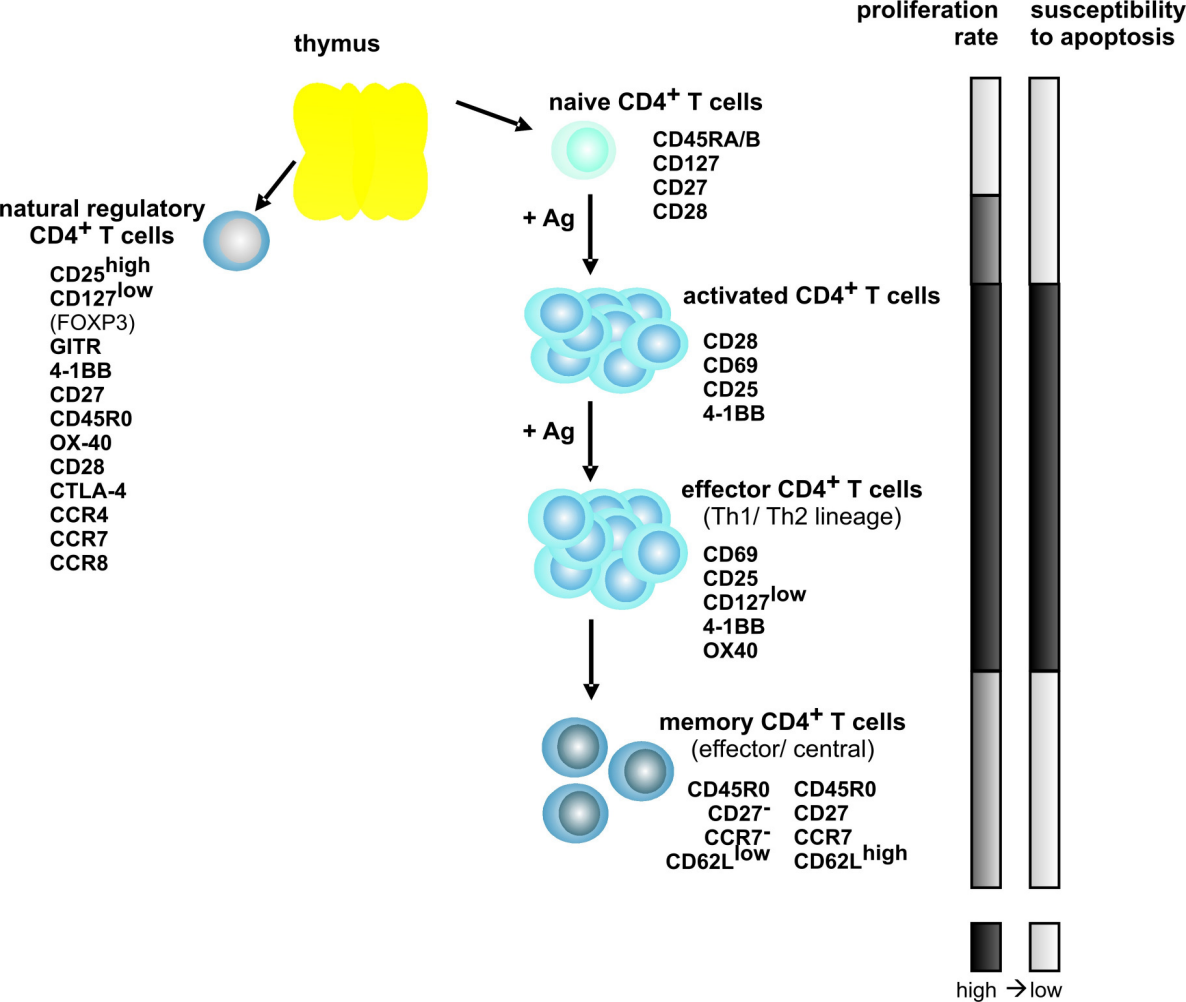
Model of CD4^+^ T cell differentiation and of the phenotype of T cell subsets. The expression of characteristic surface expression markers (except FOXP3) is exemplified. Proliferation rate and susceptibility to apoptosis of the different T cell subsets are indicated by the bars on the right. Ag indicates antigen; dark bars, high; and, bright bars, low.

The differentiation status of a T cell is important for its survival. CD4^+^ T cells, the main targets of HTLV-1-infection, are roughly grouped into different subsets (Figure 1), depending on the expression of surface markers, intracellular proteins and secretion of cytokines. Briefly, T cells are derived from progenitor cells in the bone marrow and become committed to their lineage in the thymus where they undergo positive and negative selection. Antigen recognition initiates proliferation of naïve T cells and their differentiation to activated T cells leading to changes of the phenotype. The expression of activation markers like CD69 or CD25 is induced. Depending on the nature of antigen and the inflammatory milieu, antigen-specific effector T cells are induced to differentiate into at least two functionally distinct populations of effector T cells, T helper type 1 and 2 (Th1/Th2) cells [29,30]. After pathogen elimination, most effector cells die, but some survive to form long-lived memory T cell (T mem) clones, which can be discriminated by function and surface markers into central and effector T mem [31].

Naturally occurring CD4^+^ regulatory T cells (T reg) develop either in the thymus, or they arise from mature T cells recruited to the regulatory population in the periphery [32]. They comprise less than 10% of the CD4^+^ T cell pool in human blood. Functionally, T reg actively suppress activation of the immune system and prevent pathological self-reactivity, *i.e.*, autoimmune disease [33]. Natural CD4^+^ T reg are mostly CD25 (IL2RA)^high^, CD127 (IL7R)^low^, and they express FOXP3 as well as other characteristic markers [34–36]. Different T cell subsets do not only differ in their proliferation rates, but also in their susceptibility to apoptosis. T mem clones, for example, are fast-proliferating compared to naïve T cell clones [37]. Moreover, persisting T mem clones exhibit a low susceptibility to apoptosis after clearance of antigen as the survival protein BCL-2 (B-cell lymphoma 2) is upregulated [38].

The persistence of HTLV-1 in T cell clones, which are detectable over many years, suggests that proteins mediating survival and proliferation of long-lived T cell clones could be crucial for HTLV-1 persistence and, thus, be potential targets of its oncoprotein Tax. Comparison of gene expression profiles of HTLV-1-infected/transformed cells with those of uninfected cells revealed differences in the expression pattern [39–41] including Tax-dependent changes [42,43] and genes encoding surface proteins [44]. This review aims to depict various differentially expressed surface markers of HTLV-1-transformed cells, which may be important for communication of the infected cells with their environment, thereby contributing to viral persistence, survival and longevity of HTLV-1-transformed T cell clones.

## 4. Lineage and Activation Markers of HTLV-1-Infected Cells

HTLV-1-infected cells express several lineage markers on their surface. For ATLL cells, an international consensus meeting provided phenotypic properties of ATLL cells. Typical ATLL cells are characterized by integration of the HTLV-1 provirus, their nuclei are lobulated (flower cells), and phenotypically, they resemble mature CD4^+^ T cells. ATLL cells express CD2, CD5, CD25 (IL2-RA), CD45R0, CD29 (integrin β 1), T cell receptor αβ, and HLA-DR [45]. Lack of CD7 and CD26 (dipeptidyl peptidase 4) as well as diminished expression of CD3 are further characteristics [45,46]. Most ATLL cells are positive for CD52, although some patients lack this surface marker. Both CD52 and the transferrin receptor (TFRC; CD71) are also overexpressed on HTLV-1-transformed cells [45,47]. The immunophenotype of several HAM/TSP patients compared to uninfected controls has been shown before [48].

In HTLV-1-infected cells, several immunoreceptors and activation markers are deregulated. CD45R, also known as leukocyte common antigen (LCA), is a receptor-like protein tyrosine phosphatase (protein-tyrosine phosphatase receptor type c (PTPRC)), which regulates, amongst others, src family kinases. CD45R is expressed on all nucleated cells of the hematopoietic system and plays a critical role in antigen-stimulated proliferation of T lymphocytes. CD45R0, a light molecular weight isoform of CD45R, is expressed on activated T cells and memory T cells, while CD45RA, a high molecular weight isoform, is expressed on naïve T cells [49–51]. *In vivo*, CD4^+^CD45R0^+^ T cells are the main cell type infected with HTLV-1 [52]. In ATLL, the number of naïve T cells is reduced, while the number of memory T cells is increased and correlates with HTLV-1 provirus load [53]. Moreover, the pattern of CD45R0 expression can correlate with the clinical outcome of infection as acute-type ATLL patients with CD45R0^+^ lymphocytes with intermediate expression show a better prognosis than those who lack CD45R0^+^ cells with intermediate expression [54]. In HTLV-1-transformed cell lines, the expression pattern of CD45R0 is divergent. While CD45R0 is expressed on interleukin 2(IL2)-dependent growing, Tax-low cells, it is absent on most IL2-independent growing, Tax-high cell lines [55]. To comprehensively analyze surface marker expression, we performed surface staining of several differentiation markers including CD45R0 which are typical for long-lived T cell populations and examined their expression by flow cytometry (Table 1). Our analysis included four types of HTLV-1-transformed cell lines [41,43,56–58], namely (1) HTLV-1 *in vitro*-transformed, (2) ATLL-derived, (3) HAM/TSP-derived, and (4) Tax-transformed cell lines. With regard to CD45R0, we made comparable observations in similar cell types as CD45R0 was present in all cell lines except IL2-independent cell lines (C91-PL, MT-2, HuT-102).

Among activation markers, CD80 (B7-1) and CD86 (B7-2) are upregulated on HTLV-1-infected cells [59,60]. Both are structurally similar members of the immunoglobulin superfamily expressed on a variety of hematopoietic cell types. CD80 and C86 interact with CD28 costimulatory and CTLA4 inhibitory receptors on T cells [61]. Following infection with HTLV-1, CD80 and CD86 are constitutively expressed suggesting that HTLV-1-infected CD80^+^/CD86^+^ T cells serve as antigen presenting cells, leading to a sustained proliferation of T cells [62]. In freshly-isolated PBMC from ATLL patients, both CD80 and C86 were upregulated after short-term culture and spontaneous Tax expression [63]. We confirmed consistent overexpression of CD80 on all types of HTLV-1-transformed cell lines including the Tax-transformed cell line Tesi, whereas the expression profile of the early T cell activation marker CD69 was divergent (Table 1). In ATLL patient cells, however, CD69 was not expressed, but could be induced following mitogenic stimulation [64].

## 5. Costimulatory Receptors of the Tumor Necrosis Factor Receptor (TNFR) Superfamily

Activation of T cells requires two different signals including (1) a signal provided by the T cell receptor complex after recognition of peptides presented by MHC II molecules on antigen-presenting cells, and (2), a second, costimulatory signal which is provided by CD28, a member of the immunoglobulin superfamily, upon ligation with CD80 or CD86. Generally, costimulatory signals provided by CD28 are necessary to initiate T cell activation. The same holds true for costimulatory receptors of the tumor necrosis factor (TNF) receptor superfamily, although most of them deliver their signals after CD28 [65].

The TNF receptor (TNFR) superfamily comprises three groups: (1) death domain (DD)-containing receptors, (2) decoy receptors, and (3) TNF receptor associated factor (TRAF) binding receptors including costimulatory receptors [65]. Although HTLV-1-transformed cells express receptors of all three subtypes [66–69], costimulatory TRAF binding TNFR will be focused on here due to their importance for proliferation.

TRAF binding receptors are type I transmembrane proteins that contain intracellular motifs of 4–6 amino acids which function to recruit TRAF proteins. In general, these receptors are associated with cellular activation, differentiation, and survival signaling. Upon binding of ligands, which are type II cell surface glycoproteins, signaling pathways are induced and determined by intracellular TRAFs [65,70]. Activation of the costimulatory TNF receptors OX40 (TNFRSF4) and 4-1BB (TNFRSF9), e.g., leads to activation of the NF-κB and the phosphoinositide-3-kinase (PI3K)/AKT pathways, and to increased expression of anti-apoptotic molecules including BCL-2, BCL-XL (BCL2L1) and BFL1 (BCL2A1) [70]. As a consequence, 4-1BB (TNFRS9) and OX40 (TNFRSF4) mediate survival and proliferation of long-lived T cell clones and could therefore be crucial for HTLV-1 persistence and, thus, be potential targets of its oncoprotein Tax. While being mostly absent from naïve T cells, they are present on long-lived T lymphocyte clones like T reg and T mem [35,70]. In the latter, they can augment proliferation and survival by providing anti-apoptotic signals several days after a naïve T cell encounters antigen. These signals allow continued turnover of cells and provide survival signals to prevent excessive T cell death. Consequently, the number of effector cells entering the memory pool is increased [38,70,71].

Several costimulatory receptors and ligands of the TNF family are deregulated in HTLV-1-transformed cells. gp34 was first identified in cells expressing HTLV-1 [72]. Its expression is inducible by Tax via NF-κB responsive elements in the promoter [73]. Later, gp34 was described as a type II transmembrane protein belonging to the TNF family, TNFSF4, and found to be the ligand for OX40 (OX40L) [74,75]. OX40L is expressed on normal T and B cells upon antigen stimulation while mitogens, phorbol ester, ionophores and IL-2 fail to induce OX40L in human T cells. In addition to OX40L, the receptor OX40 (TNFRSF4) is specifically upregulated in HTLV-1-infeceted cells by Tax-mediated promoter upregulation [76], suggesting an autocrine loop. In contrast to OX40L, OX40 was also present on freshly isolated ATLL cells and found on infiltrating cells of skin biopsies of ATLL patients [63,77]. Moreover, expression of OX40 increased significantly after cultured ATLL cells expressed high amounts of Tax spontaneously [63].

The costimulatory TNFR GITR (glucocorticoid-induced tumor necrosis factor receptor family-related gene; TNFRSF18; AITR) is expressed at low levels on resting T cells, but it is upregulated upon activation or antigen-stimulation of CD4^+^ and CD8^+^ T cells. On naturally occurring T reg, GITR is constitutively expressed and important for maintenance of T reg in the periphery, while it is not essential for their development [78]. Overexpression of GITR was identified in the presence of HTLV-1 using massively parallel signature sequencing [79]. In addition, a high frequency of GITR surface expression was a unique feature of all different types of HTLV-1-transformed cell lines (Table 1) and extends earlier observations of protein expression in *in vitro*-transformed cell lines [79] and of transcript expression in primary ATLL cells [80].

Although both CD40 (TNFRSF5) and its ligand CD40L can be upregulated by Tax, CD40L expression is absent in HTLV-1-transformed cell lines due to epigenetic mechanisms, but can be restored in cells from ATLL patients. This indicates, that CD40L is downregulated by distinct mechanisms in HTLV-I transformed cell lines and ATLL patients [64,81]. Upregulation of the ligand CD70 (TNFSF7, CD27L) was identified in freshly HTLV-1-*in vitro* immortalized peripheral blood mononuclear cells (PBMC) compared to proliferating T cells using gene expression arrays and Northern blot analysis [39]. Expression of CD70 protein could also be detected on HTLV-1-transformed cell lines and on fresh PBMCs from ATLL patients [44]. Thus far, CD70 expression could not be associated with survival advantages of HTLV-1-transformed cells [44]. Interestingly, the natural receptor of CD70, CD27 (TNFRSF7), is not expressed on HTLV-1-transformed cells (Table 1) ruling out a possible autostimulatory mechanism of the CD27/CD70 receptor-ligand pair.

To identify additional costimulatory receptors, which are Tax-dependently expressed, our group screened mRNA from T cells with repressible Tax expression and found that among all costimulatory receptors, transcripts of 4-1BB (TNFRSF9) were increased most strongly [43]. Upregulation of 4-1BB was a consistent feature of HTLV-1-transformed cell lines (Table 1) and was caused by efficient transactivation of the 4-1BB promoter by Tax via the NF-κB pathway. Additionally, the ligand of 4-1BB, 4-1BBL (TNFSF9) was expressed on HTLV-1-transformed cells (Table 1) suggesting auto-stimulation. In the presence of Tax, 4-1BB expression was strongly stimulated on the surface of CD4^+^ T cells isolated from HTLV-1-infected patients. [43]. Thus, the costimulatory receptor 4-1BB is a target of Tax stimulation in cultured cells and in patients, and is likely to support the survival of HTLV-1-infected T-cell clones. Taken together, the presence of several costimulatory receptors and their ligands on HTLV-1-transformed cells suggests that costimulatory signals contribute to growth and survival of the infected cell, and therefore favor a transformed phenotype.

## 6. Chemokine Receptors

Chemokine receptors belong to the family of G protein-coupled receptors (GPCRs), which contain seven transmembrane domains. Upon binding of the respective ligand, they mediate transduction of signals via intracellularly located heterotrimeric G-proteins. Several chemokines and their receptors are involved in migration of lymphocytes [82]. As ATLL is frequently accompanied by infiltrations of leukemic cells into various organs like lymph nodes or skin [83], an altered expression pattern of chemokine receptors and their ligands may be expected.

Over-expression of the secreted, anti-apoptotic chemokine I-309, as well as expression of its cognate receptor CCR8 on ATLL-derived cells, were suggested to generate an anti-apoptotic autocrine loop which could contribute to the growth of ATLL-cells [41]. CCR9, which is involved in T-cell homing to the gastrointestinal tract, was found in ATLL cells infiltrating the gastrointestinal tract and expressed on cell lines carrying HTLV-1 [84]. Chemokine receptor 7 (CCR7/EBI1/CMKBR7) is naturally expressed on central T mem [37] and required for *in vivo* function of CD4+CD25^+^ T reg [85]. Enhanced expression of CCR7 in ATLL patients correlated with lymphoid organ involvement [83]. Additionally, CCR7 expression was also a consistent feature of all HTLV-1-transformed cell lines (Table 1), although the expression levels exhibited a broader variation and lacked continuity, which may be due to receptor internalization, a characteristic of chemokine receptors. CCR7 overexpression could be an important mechanism for the establishment of a persistent viral infection of lymphoid cells which is not limited to HTLV-1 infection. Other persisting viruses such as the B-lymphotropic EBV are known to transactivate CCR7 expression via the EBV nuclear antigen 2 (EBNA2) [86]. Interestingly, besides CCR7, additional chemokine receptors including the skin homing receptors CCR4 and CCR10 are also expressed on ATLL cells from patients [87]. Among these receptors, CCR4 has been studied most intensively because of the association between predominant CCR4 expression on ATLL cells with skin involvement and unfavorable outcome [88,89], CCR4 has been successfully tested as a target for immunotherapy in ATLL in a phase I study [90,91]. Interestingly, HTLV-1-infected T cells Tax-dependently produce CCL22, the ligand of CCR4, too. Thereby, they can selectively interact with CCR4^+^CD4^+^ T cells, resulting in preferential transmission of HTLV-1 to CCR4^+^CD4^+^ T cells [92]. In conditionally Tax-expressing lymphocytes, activation of SDF-1/CXCR4 signaling correlated with Tax expression [93]. This pathway was shown to be important in other systems, too, as the use of a CXCR4 antagonist suppressed migration of cultured cells from ATLL patients and of murine lymphoblastoid cells from HTLV-I Tax transgenic mice [94]. Taken together, chemokines and their receptors are massively exploited during HTLV-1-mediated pathogenesis.

## 7. Interleukin Receptors

Tax stimulates expression of cellular interleukins and their receptors including interleukin 2 (IL2) and the alpha subunit of the IL2 receptor (IL2RA, CD25), IL13 and the receptor chains IL4RA and IL13RA, IL15 and its receptor IL15 (reviewed by [22]), as well as IL21 and the IL21 receptor (IL21R) [95]. By contrast, low IL7R (CD127) expression is, apart from ATLL-derived cell lines [96], a common feature of all types of HTLV-1-transformed cells (Table 1).

Among the interleukin receptors, IL2RA was the first cellular gene reported to be upregulated by Tax [97,98]. High expression of IL2RA is also a consistent feature of HTLV-1-transformed cell lines (Table 1). Together with the subunits IL2RB (CD122) and the common γ chain, IL2RG (CD132), IL2RA forms a functional IL2 receptor (IL2R). In addition to IL2RA, Tax stimulates the IL2 promoter [99–101] which led to the hypothesis of T cell proliferation through an autocrine IL2/IL2R loop in HTLV-1-transformed cells [22]. However, most HTLV-1/Tax-immortalized cells or ATLL-patient-derived cells in culture do not express high levels of IL2 [102] and even require exogenous IL2 for their growth [19,56,103]. Therefore, the role of an IL2/IL2R autocrine loop in leukemogenesis and transformed growth in culture remains to be determined.

Additionally, Tax also induces expression of IL15 [104], which signals through a functional IL15 receptor composed of the Tax-inducible IL15RA chain [105] and two components of the IL2 receptor, IL2RB and the common γ chain IL2RG. The existence of an IL15 autocrine loop was suggested in PBMC from HAM/TSP patients [106]. IL9, which unlike its IL9RA chain is also activated by Tax, shares the common γ chain with IL2 and IL15 receptors and functions by a paracrine mechanism in ATLL [107]. A recent study emphasized the relevance of IL2-, IL9-, and IL15-mediated signaling for HTLV-1-associated pathogenesis. *Ex vivo* spontaneous proliferation of PBMCs from ATLL and HAM/TSP was inhibited using a selective inhibitor of Jak3, which blocks signaling mediated by IL2, IL9 and IL15 [108].

The IL4/IL13 receptor complex provides stimulatory signals via the IL4RA chain. In contrast to IL4, IL13 is upregulated and secreted in HTLV-transformed cells and in cultured ATLL-cells derived from patients [109,110]. In HTLV-cells, IL13 expression is upregulated by Tax dependent on a NF-κB-responsive element in the promoter [111]. IL13 is linked to leukemogenesis, since in both Hodgkin's lymphoma cells and HTLV-1-transformed cells, it seems to act through an autocrine mechanism [22].

## 8. Adhesion Molecules on HTLV-1-Infected Cells

The Tax protein also affects T cell interactions as it stimulates the expression of adhesion molecules like the CD2 receptor CD58 (LFA-3), intercellular adhesion molecule-1 (ICAM-1, CD54) [112,113], and vascular cell adhesion molecule 1 (VCAM-1) [114]. While VCAM-1 could be detected on freshly isolated T cells from HAM/TSP patients [115], ICAM-1 and LFA-1 were downregulated on ATLL cell lines [116]. By contrast, another study showed consistent and high expression of ICAM-1 and an active form of LFA-1, which is a counter-receptor for ICAM-1, on fresh PBMC from ATLL patients. It was proposed that the proliferation of ATLL cells occurs in sequential events, including (1) homotypic and calcium-dependent adhesion through LFA-1/ICAM-1, (2) signal transduction through these adhesion molecules, (3) production of cytokines, and (4) proliferation [117]. Not only proliferation of the infected cell population, but also of uninfected cells may be regulated by adhesion molecules. In coculture experiments, irradiated or fixed HTLV-1-infected clones from HAM/TSP patients induced the proliferation of autologous, uninfected T cells dependent on CD2/LFA-3, LFA-1/ICAM1, and CD25 [118]. This hints at bystander effects of adhesion molecules on uninfected cells. In HTLV-1-infected T cells, stimulation of ICAM-1 on the cell surface in combination with intracellular Tax protein expression is sufficient to trigger polarization of the microtubule-organizing center (MTOC) at the virological synapse [119,120].

Interestingly, the HTLV-1 accessory protein p12 downregulates ICAM-1, ICAM-2 and MHC class I molecules, thereby avoiding immune recognition [121]. Additionally, the initially described capacity of p12 to increase T cell contact by clustering of lymphocyte function-associated antigen-1 (LFA-1) [122], was mapped to the viral p8 protein, which is generated from p12 by removal of an endoplasmatic reticulum retention signal. p8 also increases inter-cellular conduits thereby enhancing cellular communication and virus transmission [123,124].

Recently, the cell adhesion molecule tumor suppressor in lung cancer (TSLC1; IGSF4), a member of the immunoglobulin superfamily, was found to be overexpressed in acute-type ATLL cells [125]. Due to the ability of TSLC1 to enhance self-aggregation of ATLL cells and their adhesion to vascular endothelial cells, the authors speculated that TSLC1 may participate in tissue invasion, which is frequently found in ATLL. Function and properties of surface molecules important for adhesion, binding and entry of HTLV-1 including glucose transporter 1 (GLUT1), neuropilin 1 (NRP1) and heparan sulfate proteoglycans have recently been reviewed by Ghez *et al.* [126].

## 9. Contribution of Cell Surface Markers to Longevity and Pathogenesis

The data summarized in this review provide evidence that HTLV-1-transformed cells assume biological features of long-lived T cell clones as they express characteristic expression markers like CD4^+^CD25^+^CD45R0^+^CD127^low^4-1BB^+^GITR^+^CCR7^+^ on their surface. These differentiation markers include, amongst others, characteristic lineage markers, costimulatory receptors, chemokine receptors, interleukin receptors and adhesion molecules. Signals provided by the receptors and their ligands might contribute to survival and growth of the HTLV-1-infected cell, and thus, contribute to longevity of infected T cell clones. Moreover, the infiltrations observed in ATLL could be supported by expression of several chemokine receptors and adhesion molecules.

Expression of many of these surface markers could be attributed to expression of the viral Tax oncoprotein. However, there are differences in viral gene expression between cell lines and freshly isolated PBMC from ATLL patients. While Tax is uniformly expressed in cell lines with varying amounts [58], most freshly isolated ATLL patient cells do not express Tax spontaneously [68,127]. This can be explained by genetic modifications of Tax, DNA methylation or deletions in the 5′ LTR of the provirus resulting in silencing of viral gene expression (reviewed in [127]). Paradoxically, a persistently activated cytotoxic T lymphocyte (CTL) response to Tax, the immunodominant target that is recognized by CTL, is found in most infected patients [68,128,129] and thought to regulate viral gene expression. Removal of CD8^+^ T cells from patient samples *ex vivo* leads to spontaneous expression of Tax after short-term culture [68]. Concomitant with the loss of Tax expression in ATLL-patients, several of the markers, like 4-1BB, are, as indicated before, induced after spontaneous expression of Tax in CD4^+^ T cells of ATLL patients. Other markers, like CCR4, are expressed in ATLL patient samples independent of Tax expression [130]. It is unlikely that all markers regulated by Tax and/or involved in immune-signaling are expressed at once and at high levels in PBMC in ATLL cells as this would require permanent Tax expression in several cases and induce immune response. The individual role of each marker, its temporal regulation and fine-tuning of its expression during HTLV-1-persistence and development of pathogenesis remains to be analyzed in more detail in infected ATLL patients.

Despite several therapeutic approaches, ATLL still has a very poor prognosis due to resistance to chemotherapy. Interestingly, targeting of several of the described cellular surface proteins on malignant cells has already been used as an alternative therapeutic approach [131,132]. Monoclonal antibodies targeting IL2RA (CD25), CD2, CD52, and CCR4 were already tested in clinical trials [45]. Therefore, further investigation of the surface phenotype may elucidate novel targets for therapy of HTLV-1-associated disease.

## Figures and Tables

**Table 1.** Expression of differentiation markers on HTLV-1-transformed cells detected by flow cytometry.

**% Expression^a^**												
**Origin**	**Name**	**IL2^b^**	**PTPRC** CD45R0	**CD69**	**CD80 B7-1**	**TNFRSF18** GITR	**TNFRSF7** CD27	**TNFRSF** 4-1BB	**TNFSF9** 4-1BBL	**CCR7** EBI1	**IL2RA^c^** CD25	**IL7R** CD127
*in vitro***transformed**	**C91-PL**	no	0 +/− 0	0 +/− 0	82 +/− 17	41 +/− 3	0 +/−0	38 +/− 11	68 +/− 9	73 +/− 42	99 +/− 1	3 +/− 1
	**MT-2**	no	1 +/− 0	1 +/− 1	84 +/− 15	76 +/− 7	0 +/− 0	39 +/− 9	74 +/− 6	79 +/− 46	99 +/− 0	2 +/− 1
**ATLL-derived**	**HuT-102**	no	5 +/− 2	0 +/− 0	88 +/− 11	99 +/− 0	0 +/− 0	65 +/− 5	70 +/− 9	63 +/− 36	100 +/− 0	1 +/− 1
	**ATL-3**	40	96 +/− 1	ND^d^	74 +/− 8^c^	72 +/− 9	0 +/− 0	21 +/− 5	19 +/− 6	21 +/− 12	99 +/− 0	2 +/− 2
	**Champ**	20	88 +/− 5^c^	37 +/− 16	75 +/− 8^c^	83 +/− 8	1 +/− 1	43 +/− 4	31 +/− 5	31 +/− 18	99 +/− 1	3 +/− 1
	**JuanaW**	20	89 +/− 5	45 +/− 13	60 +/− 1^c^	92 +/− 3	0 +/− 0	18 +/− 6	24 +/− 8	55 +/− 32	98 +/− 0	2 +/− 2
	**PaBe**	20	96 +/− 3	5 +/− 1	66 +/− 13	39 +/− 0	0 +/− 0	7 +/− 3	21 +/− 3	19 +/− 11	100 +/− 1	3 +/− 2
	**StEd**	40	70 +/− 5	13 +/− 4	88 +/− 10	79 +/− 1	1 +/− 0	9 +/− 4	16 +/− 1	23 +/− 15	95 +/− 4	2 +/− 2
**HAM/TSP-derived**	**Abgho**	40	95 +/− 1	31 +/− 15	67 +/− 10^c^	71 +/− 10	1 +/− 0	26 +/− 8	18 +/− 4	27 +/− 2	95 +/− 1	4 +/− 2
	**Eva**	20	80 +/− 3	28 +/− 9^c^	75 +/− 5^c^	76 +/− 6	1 +/− 0	39 +/− 4	24 +/− 3	38 +/− 2	96 +/− 2	7 +/− 2
	**Nilu**	20	72 +/− 3	9 +/− 2^c^	45 +/− 8^c^	41 +/− 4	0 +/− 0	6 +/− 1	12 +/− 3	18 +/− 5	92 +/− 0	1 +/− 0
	**Xpos**	20	62 +/− 14	38 +/− 26^c^	60 +/− 5	91 +/− 3	1 +/− 1	31 +/− 3	16 +/− 2	60 +/− 12	99 +/− 1	5 +/− 0
**Tax-transformed**	**Tesi**	40	57 +/− 4^c^	1 +/− 1^c^	ND	93 +/− 2^c^	0 +/−0^c^	13 +/− 7^c^	15 +/− 1	9 +/− 1^c^	96 +/− 1	2 +/− 1^c^

a Values represent the mean of percent surface expression +/− SE (rounded values) as detected by flow cytometry of HTLV-1-transformed cell lines (n ≥ 3). Antibodies and protocol are given in the Appendix (Table 2);

b Cell lines were grown without interleukin 2 (no IL2) or in the presence of either 20 or 40 U/mL IL2;

c Two experiments were performed;

d ND, not done.

## Appendix

**Table 2.** Monoclonal antibodies and isotype controls used in flow cytometry.

**Antigen**	**Conjugate**	**Isotype**	**Clone**	**Origin**	**Company**
CCR7	FITC	IgG2a	150503	mouse	R&D systems, Wiesbaden, Germany
CD25 (IL2RA)	PE	IgG1	M-A251	mouse	BD, San Jose, CA, USA
CD27 (TNFRSF7)	FITC	IgG1	O323	mouse	NatuTec, Frankfurt/M., Germany
CD45RO (PTPRC)	FITC	IgG2a	UCHL1	mouse	NatuTec
CD69	PE	IgG1	L78	mouse	BD
CD80	FITC	IgG1	MEM-233	mouse	EuroBioSciences, Friesoythe, Germany
CD127 (IL7R)	PE	IgG1	hIL-7R-M21	mouse	BD
CD137 (TNFRSF9/4-1BB)	FITC	IgG1	4B4-1	mouse	AbD Serotec, Oxford, UK
CD137L (TNFSF9/4-1BBL)	R-PE	IgG1	C65-485	mouse	BD
GITR (TNFRSF 18)	FITC	IgG1	110416	mouse	R&D Systems
IgG1 isotype control	R-PE	IgG1	MOPC-21	mouse	BD
IgG1 isotype control	FITC	IgG1	MOPC-21	mouse	BD
IgG2a isotype control	FITC	IgG2a		mouse	NatuTec

IgG indicates immunoglobulin G; FITC, fluorescein isothiocyanate; PE, phycoerythrin.

### Analysis of Surface Expression by Flow Cytometry

Expression of differentiation markers was analysed by flow cytometry. Briefly, 1 × 10^6^ cells were washed in FACS-buffer (PBS without Ca^2+^ and Mg^2+^ containing 5% FCS and 0.01 % NaN_3_) and fixed in 2% paraformaldehyde in PBS. Thereafter, cells were stained with monoclonal antibodies (Table 2) and the respective isotype control antibodies for 45 min on ice. After two wash steps in FACS-buffer, fluorescence was measured on a flow cytometer (FACS Calibur, BD, San Jose, CA, USA). Data were evaluated with FCS Express version 3 [133] and the mean of % positivity of surface expression +/− standard error (SE) of three independent experiments was calculated.
